# Unilateral and Reversible Hypoglossal Nerve Palsy in Infectious Mononucleosis Syndromes: Two Rare Cases from Our Clinic

**DOI:** 10.3390/v18020200

**Published:** 2026-02-03

**Authors:** Gheorghiță Jugulete, Mădălina Maria Merișescu, Alexandra Totoianu, Mihaela Oros, Mihaela Cristina Olariu, Bianca Borcos

**Affiliations:** 1 Faculty of Dentistry, Department of Infectious Diseases, “Carol Davila” University of Medicine and Pharmacy, 050474 Bucharest, Romania; gheorghita.jugulete@umfcd.ro (G.J.); mihaela.olariu@umfcd.ro (M.C.O.); bianca-rybana.bizera@drd.umfcd.ro (B.B.); 2 National Institute for Infectious Diseases “Prof. Dr. Matei Bals”, European HIV/AIDS and Infectious Diseases Academy, No. 1 Dr. Calistrat Grozovici Street, 021105 Bucharest, Romania; alexandra.totoianu@gmail.com; 3 Physiology, Department of Preclinical Sciences, Faculty of Medicine, Titu Maiorescu University, No. 67A, Gheorghe Petrașcu Street, 3rd District, 031593 Bucharest, Romania; mihaela.oros@prof.utm.ro; 4 Ponderas Academic Hospital, No. 85A, Nicolae G. Caramfil Street, 014142 Bucharest, Romania

**Keywords:** infectious mononucleosis, hypoglossal nerve palsy, cranial neuropathy, Epstein–Barr virus, Cytomegalovirus, pediatric, cervical lymphadenopathy

## Abstract

Background and Clinical Significance: Hypoglossal nerve palsy is an uncommon neurological complication of infectious mononucleosis and is only rarely reported. Putative mechanisms include virus-triggered neuritis (Epstein–Barr virus (EBV) or Cytomegalovirus (CMV)) and/or mechanical compression related to cervical lymphadenopathy. Case Presentation: We report two children with infectious mononucleosis and transient unilateral hypoglossal nerve palsy. Case 1 was a 15-year-old boy with 7 days of fever and typical mononucleosis features who developed leftward tongue deviation accompanied by sialorrhea, dysarthria, and dysphagia. Laboratory testing showed marked hepatocellular injury and EBV-specific IgM positivity. Case 2 was a 9-year-old girl with a 24 h history of bilateral lateral cervical lymphadenopathy with overlying inflammatory signs; examination revealed rightward tongue deviation with similar associated symptoms. CMV-specific IgM antibodies were detected on serological testing. Both patients received systemic corticosteroids and empiric intravenous antibiotics, with supportive care. Hypoglossal nerve function fully recovered within 2–4 weeks of treatment initiation. Conclusions: These cases underscore that isolated hypoglossal nerve palsy may complicate EBV- or CMV-associated mononucleosis in children. Although the prognosis is generally favorable, the presentation warrants careful evaluation to exclude alternative causes of lower cranial neuropathies and close follow-up until complete neurological resolution.

## 1. Introduction

Mononucleosis-like syndromes represent a group of conditions with clinical manifestations similar to those of infectious mononucleosis but caused by various pathogens other than *Epstein–Barr virus* (EBV). In addition to EBV, *Cytomegalovirus* (CMV), HIV, *Toxoplasma gondii*, and adenoviruses may produce mononucleosis-like syndromes, characterized by fever, pharyngitis, lymphadenopathy, and atypical lymphocytosis [1,2,3].

The majority of cases of infectious mononucleosis caused by EBV or CMV follow a benign course; however, neurological complications occur in approximately 1–5% of patients and may include cranial nerve palsies, meningoencephalitis, Guillain–Barré syndrome, and peripheral neuropathies [1,2].

In adults, hypoglossal nerve palsy is most often associated with compressive lesions such as tumors or traumatic injury, while in pediatric patients infectious conditions—including infectious mononucleosis—represent important etiologic considerations [4,5]. The underlying mechanisms may involve the neurotropic potential of Epstein–Barr virus (EBV) and cytomegalovirus (CMV), or mechanical compression by enlarged lymph nodes affecting adjacent neural structures.

The hypoglossal nerve (cranial nerve XII) provides motor innervation to the tongue and is essential for chewing, swallowing, and articulation. Its nucleus is located in the dorsal medulla oblongata, and the nerve emerges as 10–15 rootlets between the pyramid and the olive. The fibers exit the posterior cranial fossa via the hypoglossal canal, located anterior to the foramen magnum. After leaving the skull base, the nerve descends in the neck deep to the sternocleidomastoid muscle, coursing between the small rectus capitis muscles and the internal carotid artery (ICA) and running medial to the glossopharyngeal, vagus, and accessory nerves. Continuing its trajectory between the ICA and the internal jugular vein, it approaches the styloid musculature, crosses the lateral surface of the external carotid artery, travels along the anterior border of the sternocleidomastoid muscle, and ultimately innervates the tongue [5].

Given its close anatomical relationships along this intricate cervical pathway, the hypoglossal nerve is vulnerable to any process that increases local pressure. These include tumors, reactive or malignant cervical lymphadenopathy, vascular complications such as Lemierre syndrome, and traumatic or iatrogenic injury, all of which may result in hypoglossal nerve palsy [5].

Hypoglossal nerve palsy is an uncommon neurological complication of infectious mononucleosis. The medical literature describes only isolated cases of hypoglossal paresis associated with acute Epstein–Barr virus (EBV) infection in both adults and children. This condition may occur unilaterally or bilaterally, typically emerging during the acute phase of the illness, and generally resolves spontaneously within weeks to months without residual functional deficits and with no reported recurrences [6,7,8].

## 2. Case Reports

### 2.1. Case 1

We report the case of a 15-year-old boy who presented with a seven-day history of fever, odynophagia, and bilateral laterocervical swelling. He had received symptomatic treatment, including antipyretics and three doses of cefixime prescribed by his family physician, with no clinical improvement. Subsequently, he developed deviation of the tongue to the left upon protrusion.

His medical history was notable for asthma under maintenance therapy and multiple respiratory allergies, while the family history was unremarkable.

**Figure 1:**
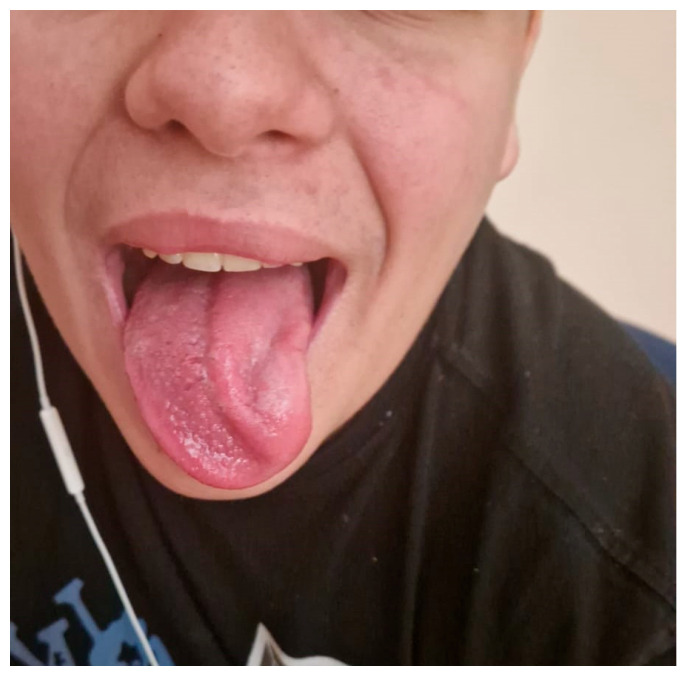
Clinical presentation of Case 1: a 15-year-old boy presenting with leftward deviation of the protruded tongue, consistent with hypoglossal nerve palsy in the context of infectious mononucleosis.

At admission, he presented with fever, slightly pale skin, and no rash. Physical examination revealed bilateral, tender, laterocervical and subangulomandibular lymphadenopathy forming neck masses; a hyperemic pharynx with whitish exudates; hypertrophic and erythematous tonsils, odynophagia, dysphagia, dysarthria and left-sided deviation of the tongue upon protrusion. The liver was palpable 1 cm below the right costal margin, and the spleen was not palpable. No other abnormalities were noted on clinical examination (Figure 1).

Neurological examination: The patient is conscious, cooperative, and oriented to time and space, with no nuchal rigidity. Cranial nerve examination is unremarkable, except for a left hypoglossal nerve dysfunction, with the tongue tip deviating to the left on protrusion and, in the resting position, lying in a near-midline position with a slight deviation to the left. Mastication, swallowing, and phonation disorders are present. No fasciculations. No motor, coordination, or sensory deficits. Deep tendon reflexes are present and symmetrical. Plantar responses are bilaterally flexor. Sphincter control is preserved. Intellectual development is normal. Significant laterocervical lymphadenopathy is noted, more pronounced on the left side. Conclusion: Left hypoglossal nerve paresis with clinical features suggestive of peripheral involvement.

Paraclinical examination: Laboratory findings (Table 1—at admission and after 5 days) showed leukocytosis (16,380 cells/mm^3^) with neutrophils 57.87%, lymphocytes 28.87%, and monocytes 12.11%. The peripheral blood smear described monocytosis. Liver enzyme levels were elevated, with ALT 501 U/L, AST 322 U/L, and GGT 167 U/L. C-reactive protein was 40 mg/L. D-dimer levels were within normal limits in this context. Serological testing was positive for EBV-specific IgM antibodies, while throat cultures for Streptococcus and Staphylococcus were negative. Laboratory parameters were re-evaluated at 28 days, at which time they had normalized.

**Figure 2:**
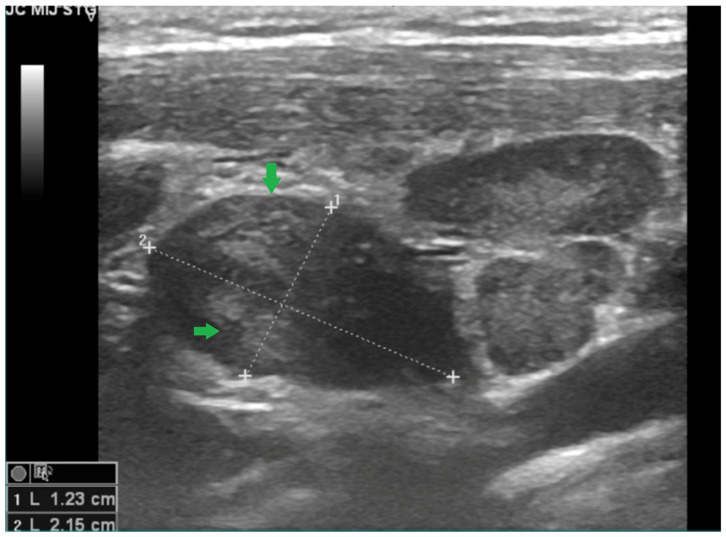
B-mode ultrasonography of the cervical soft tissues in Case 1, performed on day 7 of illness, showing an enlarged, oval lymph node with heterogeneous echotexture (arrows). Calipers indicate the maximal short- and long-axis diameters (approximately 1.23 × 2.15 cm). Image shown for illustrative purposes from a single case.

**Figure 3:**
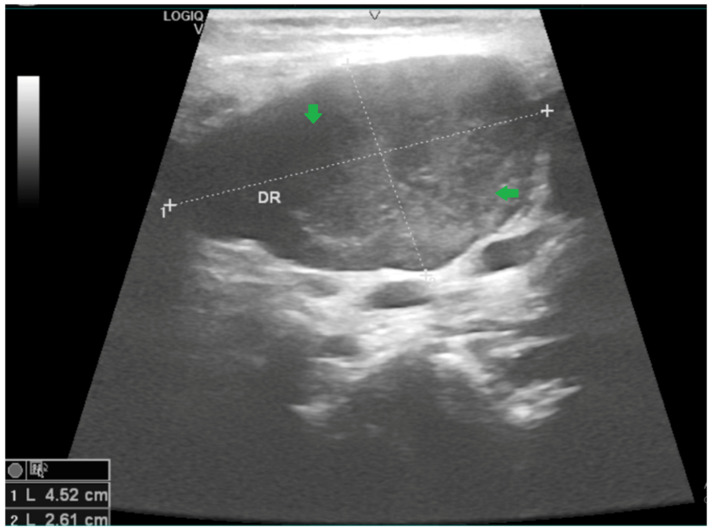
B-mode ultrasonography of the right cervical region (DR) in Case 1, performed on day 7 of illness, demonstrates a bulky, oval lymph node/mass with heterogeneous internal echoes (arrows). Calipers indicate the maximal dimensions (approximately 4.52 × 2.61 cm). Image shown for illustrative purposes from a single case.

Neck ultrasonography revealed multiple bilateral laterocervical lymph nodes, with the largest on the right side measuring 4.5 × 2.6 cm, showing ultrasound findings suggestive of suppuration (Figure 2 and Figure 3).

Contrast-enhanced MRI of the head and neck was performed, showing extensive bilateral involvement of the parapharyngeal and lateral cervical compartments, characterized by multiple enlarged lymph nodes, some demonstrating central necrotic changes and exerting mass effect on adjacent soft tissues. The brainstem—including the hypoglossal nuclei and root exit zones—shows normal morphology and signal, with no evidence of demyelination, inflammation, ischemia, or compressive pathology.

Based on the clinical and paraclinical findings, a diagnosis of Epstein–Barr virus (EBV) infectious mononucleosis associated with hypoglossal nerve (cranial nerve XII) palsy was established.

Treatment and outcome: The patient was treated with intravenous antibiotic with ceftriaxone 2000 mg daily for seven days and corticosteroid therapy with dexamethasone for seven days, with an initial dose of 16 mg/day, corresponding to 0.25 mg/kg/day, followed by a progressively tapered regimen. Under treatment, his symptoms progressively improved: fever resolved, lymphadenopathy decreased in size, and hypoglossal nerve palsy subsided gradually, with complete recovery achieved within two weeks. Gradual improvement in laboratory parameters was observed (Table 1).

### 2.2. Case 2

A 9-year-old girl presented with a 24 h history of bilateral laterocervical swelling and dysphagia. The onset was acute, and she remained afebrile throughout the course of illness.

Her medical history was notable for multiple food allergies (egg, apple, milk proteins, kiwi, carrot), manifesting as gastrointestinal symptoms and cutaneous manifestations, as well as bronchial asthma under chronic treatment. She also had a documented allergy to ceftriaxone, manifested by angioedema. Family history and living conditions were unremarkable.

At admission, the patient appeared in a moderately affected general condition, with slightly pale skin but no rash. Physical examination revealed bilateral, tender laterocervical and subangulomandibular lymphadenopathy, forming palpable cervical masses. Oropharyngeal examination showed pharyngeal hyperemia with whitish exudates and hypertrophic, erythematous tonsils, accompanied by odynophagia and dysphagia. The liver and spleen were not enlarged on examination, and the general physical assessment was otherwise normal.

Initial laboratory investigations (Table 2 at admission and after 4 days) demonstrated mild lymphopenia (2000 cells/mm^3^; lymphocytes 28.19%). Liver enzymes were mildly elevated, with ALT at 50 U/L, while AST and GGT remained within normal limits. C-reactive protein was 6.14 mg/L, and D-dimers were negative. Serological testing returned positive CMV-specific IgM antibodies, whereas throat cultures for Streptococcus and Staphylococcus species were negative.

**Figure 4:**
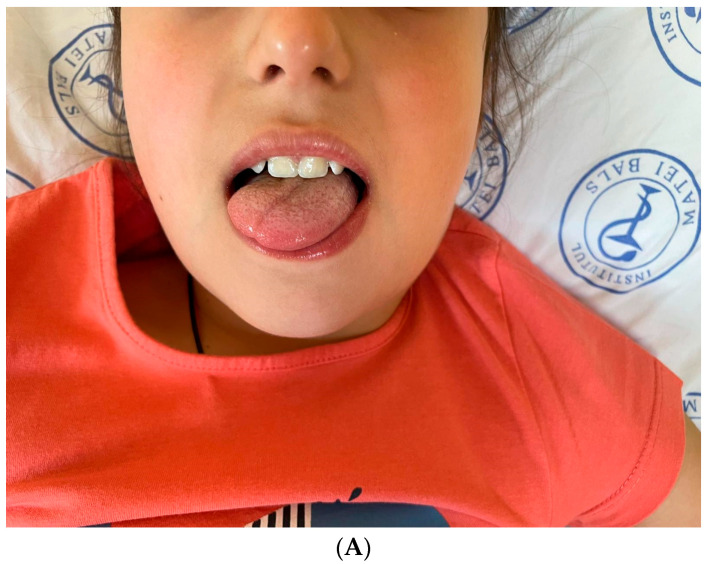

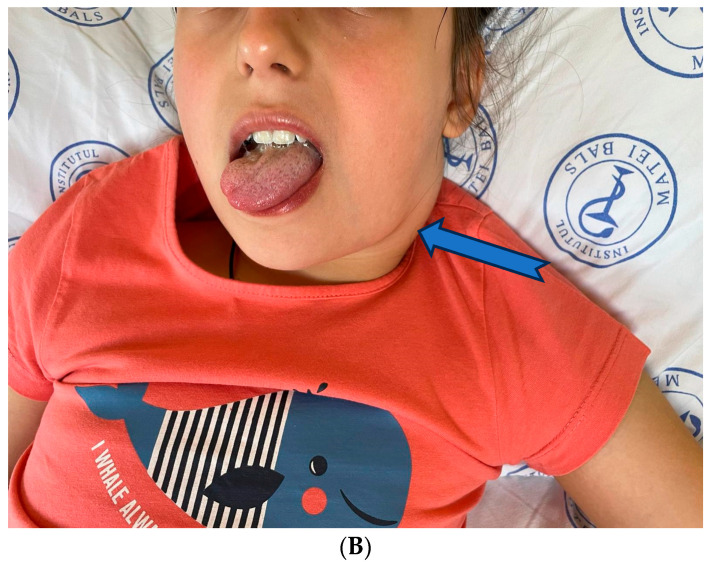
Clinical presentation of Case 2: a 9-year-old girl presenting with rightward deviation of the protruded tongue (**A**) and consistent with hypoglossal nerve palsy and pronounced cervical lymphadenopathy (**B**)—findings observed in the context of infectious mononucleosis. The arrow highlights enlarged cervical lymph nodes.

During hospitalization, 24 h after admission, the patient developed a right-sided deviation (Figure 4A) of the tongue upon protrusion, accompanied by further enlargement of the cervical lymph nodes (Figure 4B).

Neurology examination: Cranial nerves are normal except for right hypoglossal dysfunction, with tongue deviation to the right on protrusion and associated mastication, swallowing, and phonation difficulties. No fasciculations; motor, coordination, and sensory functions are intact. Conclusion: Right hypoglossal nerve paresis, clinically suggestive of peripheral involvement, in the context of infectious mononucleosis.

A cerebral and cervical CT scan was performed, which described extensive bilateral lateral cervical and parapharyngeal lymphadenopathy, with the largest lymph node measuring approximately 3.5–4.5 cm in the long axis and up to 1.5–3 cm in the short axis on the right side. Several lymph nodes show central low-attenuation areas suggestive of necrotic or suppurative transformation. The nodal enlargement causes mass effect on the adjacent parapharyngeal soft tissues, with slight medial displacement of the parapharyngeal space. No organized retropharyngeal or parapharyngeal abscess is identified.

**Figure 5:**
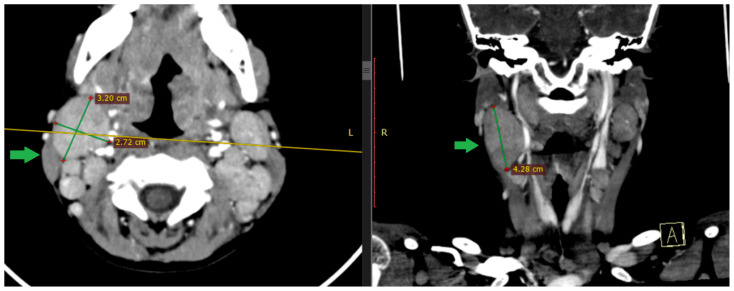
Contrast-enhanced cervical CT of Case 2, performed on day 2 of illness. **Left**: axial section showing enlarged laterocervical lymph nodes forming a conglomerate on the right side (arrow), with maximum transverse diameters of approximately 3.20 × 2.72 cm. **Right**: coronal reconstruction highlighting right laterocervical lymphadenopathy (arrow), measuring approximately 4.28 cm. Images are shown for illustrative purposes from a single case.

**Figure 6:**
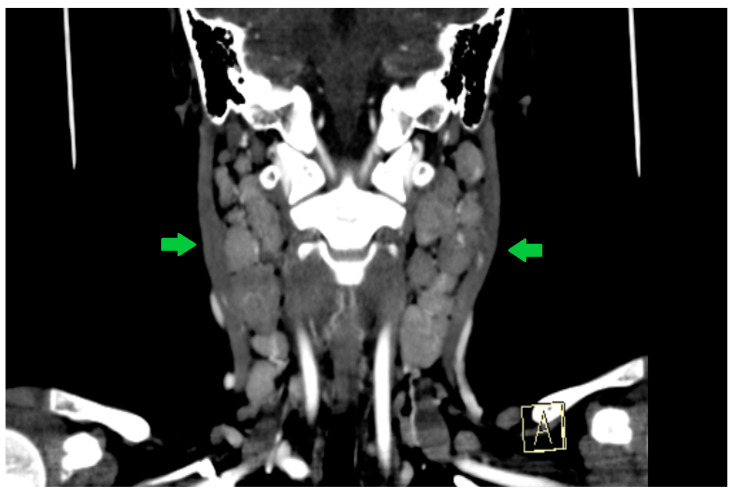
Contrast-enhanced cervical CT of Case 2, coronal reconstruction, performed on day 2 of illness, demonstrating bulky right laterocervical lymph nodes (arrows). Image shown for illustrative purposes from a single case.

**Figure 7:**
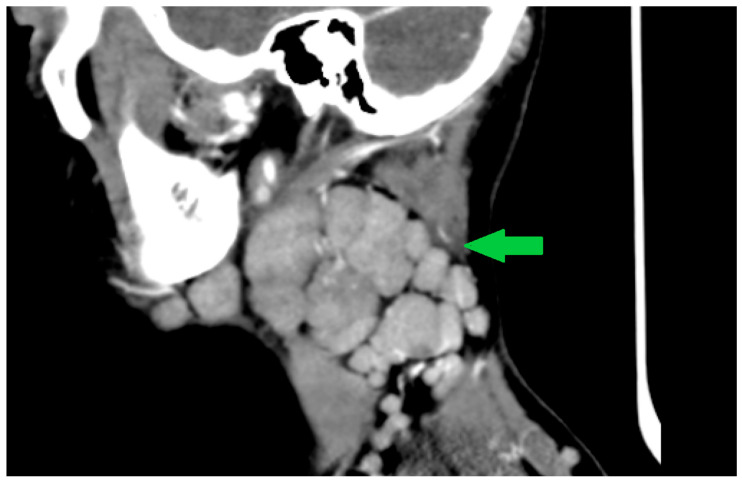
Contrast-enhanced cervical CT of Case 2, oblique sagittal section, performed on day 2 of illness, highlighting deep cervical lymphadenopathies arranged in a chain-like distribution with partial coalescence (arrows). Image shown for illustrative purposes from a single case. The arrow highlights the chain-like distribution of the cervical lymphadenopathy.

The skull base, hypoglossal canals, and intracranial segments of the hypoglossal nerves are unremarkable, with no signs of bony erosion, canal narrowing, or intracranial abnormality (Figure 5, Figure 6 and Figure 7).

Based on the clinical presentation, laboratory data, and imaging findings, a diagnosis of cytomegalovirus-induced infectious mononucleosis syndrome complicated by hypoglossal nerve (cranial nerve XII) palsy was established.

Treatment and outcome: The patient received intravenous ciprofloxacin (given the history of angioedema following cephalosporin administration) every 8 h for 7 days and corticosteroid therapy with dexamethasone for 7 days, with an initial dose of 12 mg/day, corresponding to 0.45 mg/kg/day, followed by a progressively tapered regimen. Under this treatment, her symptoms progressively improved: the lymphadenopathy gradually decreased in size, and the hypoglossal nerve palsy showed slow but progressive improvement. At 28-day follow-up, physical examination was unremarkable.

Based on the clinical presentation, alternative infectious causes of laterocervical lymphadenopathy were considered and investigated in both cases through targeted laboratory testing (serology, PCR assays, and cultures), complemented by a detailed exposure history. The work-up included investigations for *Bartonella henselae* and *Bartonella quintana*, adenovirus, enteroviruses, herpesviruses (HSV-1/2, VZV, HHV-6/7), *Borrelia* spp., human immunodeficiency virus (HIV), *Toxoplasma gondii*, and *Mycobacterium tuberculosis*. Additional pathogens potentially associated with mononucleosis-like illness or cranial neuropathies were also considered as clinically indicated, including parvovirus B19, hepatitis viruses (HAV, HBV, HCV, HEV), influenza viruses, SARS-CoV-2, *Mycoplasma pneumoniae*, and *Treponema pallidum*. Common pyogenic pathogens associated with tonsillitis were also considered; however, the available clinical and microbiological findings did not support these etiologies(Table 3). In addition, laboratory analyses together with imaging studies excluded neoplastic causes (such as lymphomas) and complications, including thrombosis (Lemierre syndrome).

## 3. Discussion

In our series, we identified unusual cases of hypoglossal nerve palsy in pediatric patients, a clinical presentation that is only rarely reported in the literature. A particularly distinctive feature of our report is the absence of previously published, objectively confirmed cases of hypoglossal nerve palsy secondary to *Cytomegalovirus* (CMV) infection. In our CMV-associated case, the clinical course was characterized by a rapid onset, with acute local inflammatory and infectious changes and a tendency toward lateral cervical abscess formation, followed by a more protracted recovery phase. Despite the pronounced local involvement, systemic manifestations were minimal: the patient remained afebrile, and routine laboratory parameters were within normal limits.

Both patients had a personal history of atopy and multiple allergies; however, this association appears incidental. Atopic or allergic status is not recognized as a risk factor for cranial neuropathies in the context of common viral infections. Moreover, IgE-mediated hypersensitivity does not imply impaired cellular immunity and therefore does not predispose individuals to more severe CMV or *Epstein–Barr virus* (EBV) infections, nor to neurological or neuritic complications.

A review of the available literature suggests that the etiological spectrum of hypoglossal nerve palsy differs substantially between adults and children. While most adult cases are noninfectious, pediatric cases are predominantly infectious. Among the 27 pediatric cases reported to date, infections represented the most frequent etiology, followed by inflammatory conditions. Hematologic or oncologic disorders, trauma, iatrogenic causes, and post-vaccination events—including those occurring after influenza vaccination—were reported only rarely. In the specific setting of infectious mononucleosis, nine pediatric cases have been described [6,7,8], typically presenting as unilateral, isolated, and reversible hypoglossal nerve palsy. In contrast, cases associated with Lemierre syndrome generally followed a more severe and complex clinical course. Other infectious etiologies, such as tuberculosis, may present with similar neurological findings; however, these cases usually have a more insidious onset and a more pronounced systemic involvement [5,8].

Accurate diagnosis relies heavily on imaging studies. Soft-tissue ultrasound, computed tomography (CT), and magnetic resonance imaging (MRI) are essential to exclude Lemierre syndrome and other local structural pathologies, including tumors or inflammatory masses [5]. In both patients, radiological imaging and laboratory investigations, including D-dimer levels, were unremarkable, effectively excluding venous thrombosis and Lemierre syndrome. From a differential diagnostic standpoint, laterocervical lymphadenopathy accompanied by a mononucleosis-like syndrome warrants consideration of a broad range of infectious agents. Accordingly, in both patients we pursued targeted investigations (serology, PCR assays, and cultures) for several relevant pathogens, including *B. henselae*/*B. quintana*, adenovirus, enteroviruses, herpesviruses (*HSV-1/2*, *VZV*, *HHV-6/7*), *Borrelia* spp., *HIV*, *M. tuberculosis*, and *T. gondii* [6,7,8,9]. In addition, other viral etiologies that may clinically resemble mononucleosis—*Parvovirus B19* hepatitis viruses (*HAV*, *HBV*, *HCV*, *HEV*), influenza viruses, and *SARS-CoV-2*—were considered, as were selected infections reported in association with cranial neuropathies (e.g., *M. pneumoniae* and *T. pallidum*), when clinically appropriate. Although pyogenic bacteria typically implicated in tonsillitis were also taken into account, the overall clinical evolution and available microbiological data did not support a bacterial cause in either case. Neoplastic processes were considered unlikely based on the presence of an inflammatory clinical syndrome and radiological findings that were not suggestive of malignancy. Other causes of sepsis secondary to bacterial infection were excluded through comprehensive laboratory evaluation [9,10].

Management in most published cases has been primarily supportive. The role of corticosteroid therapy remains controversial: while some authors have reported the use of steroids for their anti-inflammatory effects on neuritis and compressive lymphadenopathy, others have favored conservative management alone. Given the limited number of reported cases, firm treatment recommendations cannot yet be established. Nevertheless, the majority of pediatric cases described in the literature demonstrate complete recovery, although the time to restitutio ad integrum varies. In cases of hypoglossal nerve palsy secondary to infectious mononucleosis, recovery typically occurs within several weeks to a few months [5,10,11].

Certain clinical features may suggest an atypical or complicated course and should prompt further diagnostic evaluation. These include persistent or progressive neurological deficits, incomplete recovery after several months, bilateral cranial nerve involvement, rapidly progressive symptoms, and the presence of additional neurological manifestations such as severe headache, altered mental status, or signs of meningoencephalitis. Systemic features—including airway compromise, cytopenias, hepatosplenomegaly, or prolonged fever—should also raise concern [8,9,10,11,12]. In such situations, alternative etiologies, including neoplastic, vascular, traumatic, bacterial, or fungal causes, must be carefully excluded [13].

Recurrence should also be considered, either in the setting of repeated local inflammatory episodes (e.g., cervical lymphadenitis) or following infection with neurotropic viruses (including enteroviruses, herpesviruses, adenoviruses, influenza virus, and measles virus), as well as in the context of CMV reactivation. Nevertheless, the extremely limited number of pediatric reports precludes firm conclusions. Importantly, in immunocompetent children, recurrence does not seem to represent a typical feature. To date, recurrent hypoglossal nerve palsy secondary to infectious mononucleosis has not been reported, and the available cases generally describe a favorable clinical course without relapse [8,9,10,11].

## 4. Conclusions

Hypoglossal nerve palsy represents a rare but clinically meaningful manifestation within the spectrum of pediatric infectious mononucleosis and may occur in association with either EBV or CMV infection. Our two cases illustrate that an apparently isolated unilateral hypoglossal deficit can present with concerning functional symptoms (dysarthria, dysphagia, and sialorrhea) and may raise important diagnostic dilemmas. Although the use of systemic corticosteroids in infectious mononucleosis remains controversial, neurological recovery was observed following their administration in our patients. Given that spontaneous resolution is common, a causal relationship between corticosteroid therapy and clinical improvement cannot be established. At one month after discharge, tongue mobility had fully normalized, laboratory abnormalities had resolved, and both children remained clinically stable under follow-up. Given the broad differential diagnosis of lower cranial neuropathies, careful clinical assessment and targeted investigations are essential to exclude alternative structural, vascular, inflammatory, or neoplastic causes, particularly in the presence of marked cervical lymphadenopathy or persistent symptoms. Overall, available pediatric reports suggest a favorable prognosis with full recovery, typically within weeks; however, the limited number of published cases limits firm conclusions regarding optimal management and prognostic factors. Recurrence in immunocompetent children does not appear to be a characteristic feature, and, to date, recurrent hypoglossal nerve palsy specifically secondary to infectious mononucleosis has not been reported. Nonetheless, structured follow-up until complete neurological resolution remains advisable to document recovery, detect atypical evolution, and ensure comprehensive patient care.

## Figures and Tables

**Table 1:** Relevant diagnostic laboratory tests for Case 1 on hospital days 1 and 5 and on day 28 at follow-up evaluation.

Hospital Day	1	5	28 (Follow-Up)	Normal Values
WBC (mm^3^)	16,380	16,770	5990	4500–13,500
Neutrophils (mm^3^)	9480	9070	2430	1800–8000
Lymphocytes (mm^3^)	4570	5360	3110	3000–9500
Monocytes (mm^3^)	1980	1900	240	0.0–0.7
ALT (U/L)	501	194	33	24–45
AST (U/L)	322	84	20	22–49
GGT(U/L)	167	109	14	12–43
CRP (mg/L)	40	22	3	0–3
D-Dimers (mg/L)	0.55	0.3	−	<0.25
IgM Epstein–Barr ELISA	positive		positive	

“−” No sample was collected.

**Table 2:** Relevant diagnostic laboratory tests for Case 2 on hospital days 1 and 4 and on day 28 at follow-up evaluation.

Hospital day	1	4	28 (Follow-Up)	Normal Values
WBC (mm^3^)	9020	12,060	8090	4500–13,500
Neutrophils (mm^3^)	5840	6490	4960	1800–8000
Lymphocytes (mm^3^)	2000	4200	2210	3000–9500
Monocytes (mm^3^)	0.47	1230	660	0.0–0.7
ALT (U/L)	50	−	45	24–45
AST (U/L)	45	−	35	22–49
GGT(U/L)	28	−	14	12–43
CRP (mg/L)	6.14	10	2	0–3
D-Dimers (mg/L)	0.5	0.2	−	<0.25
IgM Cytomegalovirus ELISA	−	positive	positive	
IgG Cytomegalovirus ELISA	−	negative	borderline positive	

“−” No sample was collected.

**Table 3:** Evaluation of alternative infectious causes of laterocervical lymphadenopathy in both cases. Time of testing refers to days after hospital admission. IGRA, interferon-gamma release assay; PCR, polymerase chain reaction; BCG, Bacillus Calmette–Guérin vaccine.

Pathogen Considered	Diagnostic Approach	Specimen Type	Timing of Testing	Case 1	Case 2	Relevant Anamnesis
*B. henselae*/*B. quintana*	Serology	Blood	Day 1	Negative	Negative	C1, C2: No cat/dog contact
*Herpesviruses*	Serology	Blood	Day 1	Negative	Negative	
(*HSV-1/2*, *VZV*, *HHV-6/7*)						
*Enterovirus*	Serology	Blood	Day 1	Negative	Negative	
*Adenovirus*	PCR	Respiratory sample	Day 1	Negative	Negative	
*SARS-CoV-2*	PCR	Respiratory sample	Day 1	Negative	Negative	
*Influenza viruses*	PCR	Respiratory sample	Day 1	Negative	Negative	
*M. pneumoniae*	PCR	Respiratory sample	Day 1	Negative	Negative	
*Parvovirus B19*	Serology	Blood	Day 1	Negative	Negative	
*Borrelia* spp.	PCR	Blood	Day 1	Negative	Negative	C1, C2: No tick contact/exposure or prior tick bite was documented in either case
*T. gondii*	Serology	Blood	Day 1	Negative	Negative	
*M. tuberculosis*	IGRA	Blood	Day 1	Negative	Negative	C1, C2: No TB contact; C1, C2: BCG-vaccinated
*HIV*	Serology	Blood	Day 1	Negative	Negative	
*Hepatitis viruses* (*HAV*, *HBV*, *HCV*, *HEV*)	Serology	Blood	Day 1	Negative	Negative	
*T. pallidum*	Serology	Blood	Day 1	Negative	Negative	
*Pyogenic tonsillitis pathogens*	Culture	Throat swab	Day 1	Negative	Negative	

## Data Availability

The original contributions presented in this study are included in the article. Further inquiries can be directed to the corresponding author.

## References

[1] Luzuriaga K., Sullivan J.L. (2010). Infectious mononucleosis. N. Engl. J. Med..

[2] Sylvester J.E., Buchanan B.K., Silva T.W. (2023). Infectious mononucleosis: Rapid evidence review. Am. Fam. Physician.

[3] Ebell M.H., Call M., Shinholser J., Gardner J. (2016). Does this patient have infectious mononucleosis? The Rational Clinical Examination systematic review. JAMA.

[4] Węgiel A., Zielińska N., Głowacka M., Olewnik Ł. (2024). Hypoglossal nerve neuropathies—Analysis of causes and anatomical background. Biomedicines.

[5] Scannapiecoro C., Indolfi G., Temperino V., Trapani S. (2025). Hypoglossal nerve palsy in infectious mononucleosis and *Fusobacterium necrophorum* tonsillitis: A case report and literature review. Ital. J. Pediatr..

[6] Merisescu M.M., Luminos M.L., Pavelescu C., Jugulete G. (2023). Clinical features and outcomes of the association of co-infections in children with laboratory-confirmed influenza during the 2022–2023 season: A Romanian perspective. Viruses.

[7] Jugulete G., Merisescu M.M., Bastian A.E., Zurac S., Stoicescu S.M., Luminos M.L. (2018). Severe form of A1H1 influenza in a child—Case presentation. Rom. J. Leg. Med..

[8] DeSimone P.A., Snyder D. (1978). Hypoglossal nerve palsy in infectious mononucleosis. Neurology.

[9] Lazar M., Moroti R., Barbu E.C., Chitu-Tisu C.E., Tiliscan C., Erculescu T.M., Rosca R.R., Frasila S., Schmilevschi E.T., Simion V. (2024). The Impact of HIV on Early Brain Aging—A Pathophysiological (Re)View. J. Clin. Med..

[10] Parano E., Giuffrida S., Restivo D., Saponara R., Greco F., Trifiletti R.R. (1998). Reversible palsy of the hypoglossal nerve complicating infectious mononucleosis in a young child. Neuropediatrics.

[11] Johns M.M., Hogikyan N.D. (2000). Simultaneous vocal fold and tongue paresis secondary to Epstein–Barr virus infection. Arch. Otolaryngol. Head Neck Surg..

[12] Pérez-Pérez J. (2011). Isolated palsy of the hypoglossal nerve complicating infectious mononucleosis. Infection.

[13] Jugulete G., Merisescu M., Bastian A.E., Luminos M. (2017). Clinical Aspects and Medico-Legal Implications of Purpura Fulminans in Children. Rom. J. Leg. Med..

